# An Unexplored Equity Factor During Pre-Implementation of Contingency Management: Differential Beliefs and Attitudes by Providers’ Ethnicity

**DOI:** 10.21203/rs.3.rs-2719994/v1

**Published:** 2023-04-18

**Authors:** Oladunni Oluwoye, Douglas L. Weeks, Michael G. McDonell

**Affiliations:** Elson S. Floyd College of Medicine at Washington State University

**Keywords:** Contingency management, ethnicity, implementation, providers, substance use treatment

## Abstract

**Background:**

Although considered one of the most effective interventions for substance use disorders (SUD), the widespread uptake of contingency management (CM) has remained limited. Prior studies at the provider-level have explored beliefs about CM among SUD treatment providers and have tailored implementation strategies based on identified barriers and training needs. However, there have been no implementation strategies that have actively sought to identify or address potential differences in the beliefs about CM that could be influenced by the cultural background (e.g., ethnicity) of treatment providers. To address this knowledge gap, we examined beliefs about CM among a sample of inpatient and outpatient SUD treatment providers.

**Methods:**

A cross-sectional survey of SUD treatment providers was completed by 143 respondents. The survey asked respondents about their attitudes toward CM using the Contingency Management Beliefs Questionnaire (CMBQ). Linear mixed models were used to examine the effect of ethnicity on CMBQ subscale (general barriers, training-related barriers, CM positive-statements) scores.

**Results:**

Fifty-nine percent of respondents self-identified as non-Hispanic White and 41% as Hispanic. Findings revealed that SUD providers who identified as Hispanic had significantly higher scores on the general barriers (p < .001) and training-related barriers (p = .020) subscales compared to the non-Hispanic White SUD providers. Post-hoc analyses identified differences in the endorsement of specific individual scale items on the general barriers and training-related subscales.

**Conclusions:**

Dissemination and implementation strategies for CM among treatment providers need to consider equity-related factors at the provider-level that may be associated with the adoption and uptake CM.

## Introduction

Contingency management (CM) is an evidence-based intervention for substance use disorders (SUD) that provides positive reinforcement (e.g., prizes, vouchers, gift cards) for targeted behaviors such as abstinence or the reduction of substance use (Rash et al., 2017). Multiple studies have demonstrated the effectiveness of CM for SUD in community-based settings (McPherson et al., 2018; Petry et al., 2012; Prendergast et al., 2006). CM has also been culturally adapted to meet the treatment needs of specific ethnoracial groups (e.g., American Indian/Alaska Native) and has been effective in reducing substance use among ethnoracial minorities (Barry et al., 2009; Hirchak et al., 2018; Montgomery et al., 2015). Although previous research has demonstrated the effectiveness of CM in multiple practice settings, the widespread uptake of CM has been slow.

In the uptake schema, the pre-implementation phase is characterized as the work needed to understand organizational and provider readiness (Ellis et al., 2020; Simpson, 2002). Among providers, readiness includes understanding, knowledge, and beliefs about an intervention (Ball et al., 2002; McGovern et al., 2004). Treatment providers’ beliefs and attitudes towards CM have been well documented and are an important individual-level factor that can impede or facilitate implementation (Oluwoye et al., 2019). Some studies have found negative attitudes towards the philosophical underpinnings, operational cost, lack of training, and limited knowledge about CM as barriers among SUD treatment providers (Benishek et al., 2010; Ducharme et al., 2010; Kirby et al., 2006). Further, certain provider characteristics, such as educational attainment and years of experience, have also been shown to influence beliefs about CM (Kirby et al., 2006; Srebnik et al., 2013). Based on what is known about providers’ beliefs and attitudes towards CM, implementation strategies have been developed to include pragmatic training components to target many of these factors, such as lack of training (e.g., CM delivery) and limited knowledge (e.g., theoretical underpinnings of CM) (Oluwoye et al., 2019). However, many of these earlier studies examining beliefs and attitudes toward CM and assessing implementation strategies have not considered equity-related factors such as the race and ethnicity of providers. It is, therefore, unknown whether this specific provider-level characteristic affects beliefs and attitudes toward CM which will subsequently impact implementation success.

In other behavioral health fields, there has been a few studies that have found an association between the race and/or ethnicity of providers and attitudes towards the general use of evidence-based interventions. For instance, Aaron and colleagues’ findings suggest that Black and Hispanic providers report more negative attitudes toward evidence-based interventions compared to White providers (Aarons et al., 2012, 2010). In contrast to these studies, one study found that Hispanic providers reported more positive attitudes toward evidence-based interventions in community mental health settings (Ramos et al., 2020). To our knowledge no studies have explored the relationship between providers’ race and/or ethnicity and their beliefs about CM, in particular. Thus, the purpose of this study was to conduct a survey to characterize beliefs about CM among a racially/ethnically diverse sample of SUD treatment providers in community-based settings.

## Methods

### Participants and procedures

Potential participants were eligible if they were 18 years of age and older, self-identified as currently or previously employed as a provider in an addiction treatment clinic, and reported English language fluency. Potential participants did not need to have prior experience with CM to complete the online survey. Between January 2019 and June 2019, a total of 203 potential participants responded to a targeted email disseminated to professional groups focused on SUD and addiction (i.e., Addiction Drug and Alcohol Institute), who were asked to also share it with other networks. Of those who initially responded, 162 respondents met inclusion criteria, with 143 providing responses on the CMBQ, for a participation rate of 88.3% (The American Association for Public Opinion Research., 2016).

To facilitate survey completion, a Research Electronic Data Capture (REDCap)-based electronic survey was used to capture responses from eligible participants and the survey link was embedded in the email distributed to listservs. The result was an “opt-in” nonprobability sample based on participants recruited through the professional group listserv sampling frame. This study was considered exempt by the university’s Institutional Review Board and participants were asked to read and download informed consent forms, and indicate whether they would like to continue to the survey. At the end of the survey, participants were asked to provide their email address to receive a $15 e-gift card for survey completion. The survey was closed after six-months of recruitment and all data were maintained in a secure electronic database.

## Measures

### Contingency Management Beliefs Questionnaire

The 32-item Contingency Management Beliefs Questionnaire (CMBQ) was used to assess the level of influence each item has on providers’ decision to adopt the use of CM on a five-point Likert scale (0 = no influence at all; 1 = very little influence; 2 = some influence; 3 = strong influence; 4 = very strong influence). Exploratory and confirmatory factor analysis of CMBQ item scores suggests a stable and reliable three factor structure forming three subscales totaling 32 items: General Barriers (17 items), Training-related Barriers (4 items), and CM-supportive Statements (11 items) (Rash et al., 2012). The general barriers subscale included items related to time and cost demands of implementing CM, as well as clinical concerns. Training-related barriers pertained to lack of training opportunities and qualified supervision, as well as concerns about organizational support. The CM-supportive statements related to the perceived benefits of CM.

### Demographics

Data were also collected on key demographic characteristics, such as age, gender (male, female, transgender, or nonbinary of which respondents only selected male or female thus creating a binary variable), race (White, Black/African American, Asian, American Indian/Alaska Native, Native Hawaiian or Pacific Islander, Other), ethnicity (non-Hispanic or Hispanic), licensed mental health counselor (LMHC) credential status (yes/no), and graduate degree status (yes/no).

### Data analysis

Among all respondents, 49% self-identified as non-Hispanic White, 34% as Latinx, 7% as Black, and 10% as an ethnoracial minority. Based on the sparse representation of races and ethnicities other than non-Hispanic White and Hispanic ethnicity, we limited our analyses to these two groups in order to avoid error in interpretation from very small subsamples of other races. Demographic comparisons among non-Hispanic White and Hispanic respondents were appropriate to the scale of measurement: age was compared with independent sample t-tests with all other demographic variables analyzed in two-way contingency table analyses using exact tests to assess for significant differences in proportions by ethnicity. For inferential testing, CMBQ subscale sores were converted to the mean scale score by summing scores for all items completed within a scale and dividing by the number of items completed in the scale. Averaging standardized scales (composed of different numbers of items) for comparison and compensated for the limited amount of non-response within a few respondents, as detailed in the Results section.

A linear mixed model was used to examine the effect of ethnicity on mean CMBQ subscale scores. Ethnicity was modeled as a binary fixed factor with the each CMBQ subscale scores, representing levels of a second fixed repeated measures factor - scale. The ethnicity and scale interaction term was also modeled as the primary parameter of interest in order to evaluate whether scores on each subscale differed among non-Hispanic White and Hispanic respondents. The initial fully specified model included all demographic variables that differed significantly by ethnicity in univariate analyses as covariates (i.e., age, gender, credential status, graduate degree status). A second, more parsimonious, model was developed that removed gender and credential status from the model as these were not significant as covariates in the fully specified model (*p* = .941 and *p* = .322, respectively). Graduate degree status and age were maintained as covariates in the second model. Model fit to the data improved in the second model based on a reduction in Akaike’s Information Criterion. Both models employed random intercepts for respondents to account for correlation between CMBQ subscales, restricted maximum likelihood estimation, and modeled covariance structure as a scaled identity matrix; both models converged under these criteria.

A significant ethnicity by CMBQ subscale interaction was followed up with simple main effects tests to determine which scales differed by ethnicity. Post-hoc analyses were conducted on CMBQ subscales that differed by ethnicity using a scale-item by scale-item follow up 2 by 2 (ethnicity by dichotomized item scale score [‘some to very strong influence’ vs. ‘very little to no influence’]) exact tests to explore differences in specific attitudes toward CM among Hispanic and non-Hispanic White respondents. All analyses used two-sided type I error rates of *p* < .05, and were conducted with SPSS, v. 28.0.

## Results

### Participant characteristics

Overall (N = 143), the mean age was 41.0 years, 59% (n = 85) self-identified as non-Hispanic White and 41% (n = 58) as Hispanic. As seen in Table 1, non-Hispanic White respondents were significantly older and had significantly more female providers than participants who identified as Hispanic. A significantly higher proportion of Hispanic respondents were LMHC credentialed, while a significantly higher proportion of non-Hispanic White respondents had graduate degrees. The majority of respondents in both groups worked in addiction or mental health agencies, and most worked in outpatient settings.

### Differences in CMBQ subscale scores by ethnicity

Linear mixed modeling of CMBQ subscale scores revealed significant main effects by ethnicity (*p* = .001) and the repeated measures scale variable (*p* < .001), after adjustment for age and graduate degree status. These main effects were superseded by a significant ethnicity by CMBQ subscale interaction (*p* < 001). Simple main effects testing revealed significantly higher general barriers scale scores (*p* < 001) and training-related barriers scale scores (*p* = .020) among Hispanic respondents compared to non-Hispanic White respondents. Scores on the CM-Supportive Statement subscale did not differ by ethnicity (*p* = .853). Interaction means derived from linear mixed modeling are displayed in Table 2.

### Differences in Individual Items on General and Training-Related Barriers Subscales

For each of the 17 items in the General Barriers subscale, significantly higher proportions of Hispanic respondents endorsed “some to very strong influence” compared to non-Hispanic White respondents (all *p*-values ≤ .036). Items with the greatest differences in endorsement among groups (> 40% difference in group proportions) included external barriers (e.g., clients already abstinent so don’t need CM and clinics prevent urine screening) and internal barriers (e.g., provider finding CM distasteful and clinical experience more important than research evidence). Differences in endorsement of individual subscale items are displayed in Table 3.

On the Training-Related Barriers subscale, significantly higher proportions of Hispanic respondents endorsed “some to very strong influence” than non-Hispanic White respondents need for more CM training (*p* = .023). Similarly, a significantly larger proportion of Hispanic respondents felt their agencies/administrations posed barriers to provision of CM (*p* = .005) relative to their non-Hispanic White counterparts.

## Discussion

Findings from our study revealed valuable insights into the variability of attitudes among providers of different ethnicities towards CM as an evidence-based intervention for SUD. Although there has been limited CM research intentionally focusing on equity factors (e.g., ethnicity) at the provider-level, our findings align with prior work in other areas that have signaled the potential importance of providers’ ethnicity on beliefs and attitudes (Aarons et al., 2012, 2010; Ramos et al., 2020), which is key to designing and refining implementation strategies.

Broadly, understanding providers’ beliefs and attitudes during the pre-implementation phase is linked to the successful adoption and uptake of an intervention. This also includes identifying whether there are gaps or differences among providers, so that implementation strategies are tailored appropriately. Previous studies have cited that failure to adequately understand providers’ attitudes can hinder the successful implementation of an intervention (Ellis et al., 2020). A single study examining ethnoracial differences in the adoption of CM reported lower CM adoption rates among SUD providers who identified as an ethnoracial minority than among non-Hispanic White providers (Helseth et al., 2018). Our findings, along with limited prior work, collectively demonstrates that it is not enough to simply tailor implementation strategies to fit within the practice context. Instead, our results support the need to tailor implementation strategies for ethnoracial characteristics of SUD providers. Because few studies have examined equity-factors beyond the client level, further research is needed to determine how to improve uptake and use of evidenced-based interventions among ethnoracially diverse providers.

The field of implementation science has more recently called for equity-focused practices in the design and roll-out of strategies (Baumann and Cabassa, 2020; Shelton et al., 2020). Notwithstanding, there has been limited direction on how implementation strategies that include training and supervision can be tailored. For example, to moderate negative attitudes towards CM, it could be that evidence surrounding effectiveness of CM for initiating abstinence among ethnoracial minorities needs to be further highlighted in initial training. Storytelling videos that capture client and provider experiences engaging in a CM program, implemented in ethnoracially-specific settings could also be incorporated and widely disseminated among providers. During the pre-implementation phase, community participatory approaches that include providers as stakeholders should be incorporated to align CM practices with cultural values, which has been shown to be useful in Tribal communities (Hirchak et al., 2018).

Among study limitation is the use of a convenience sample of SUD providers. Based on this, caution should be used when generalizing to other SUD treatment providers. Further, the small number of respondents from Black, American Indian/Alaska Native, and Asian respondents suggests that these results should not be extended to other races. As such future research should examine differences in attitudes and beliefs about CM in a more diverse sample of SUD treatment providers.

## Conclusions

This study highlights the need for consideration of Hispanic ethnicity as an equity factor at the provider-level, which has not been previously acknowledged during the pre-implementation phase. Understanding the possible influence of providers’ ethnoraical background on the perceived advantages and disadvantages of CM is fundamental to developing equity focused implementation strategies that may aid the adoption of CM among SUD treatment providers.

## Figures and Tables

**Table 1:** Demographic Characteristics of Respondents

Variable	Overall (N = 143)	Non-Hispanic White (N = 85)	Hispanic (N = 58)	*p*
**Age Mean (SD)**	41.0 (11.6)	43.6 (12.5)	37.1 (8.9)	< .001
**Gender %(N)**				< .001
Male	54.5% (78)	42.4% (36)	72.4% (42)	
Female	45.5% (65)	57.6% (49)	27.6% (16)	
**Education %(N)**				
Graduate degree	37.1% (53)	50.6% (43)	17.2% (10)	< .001
**Agency Type %(N)**				.293
Inpatient	20.3% (29)	23.5% (20)	15.5% (9)	
Outpatient	79.7% (114)	76.5% (65)	84.5% (49)	
**Credentials %(N)**				
LMHC^1^ Credential	40.6% (58)	18.8% (16)	72.4% (42)	< .001
ABPP^2^ Credential	10.5% (15)	9.4% (8)	12.1% (7)	.782
LICSW^3^ Credential	8.4% (12)	8.2% (7)	8.6% (5)	.999
**Addiction/Mental Health Agency**	94.4% (135)	90.6% (77)	100% (58)	.052

1 Licensed Mental Health Counselor

2 American Board of Professional Psychology

3 Licensed Independent Clinical Social Worker

**Table 2:** Group means derived from linear mixed modeling for each Contingency Management Beliefs Questionnaire (CMBQ) scale. 95% confidence intervals are in parentheses.

CMBQ Subscales	Non-Hispanic White (n = 85)		Hispanic (n = 58)	
	M	95% CI	M	95% CI
General Barriers	2.78	(2.64, 2.93)	3.41	(3.24, 3.59)
Training-Related Barriers	3.17	(3.03, 3.32)	3.49	(3.27, 3.62)
CM-Supportive Statements	3.35	(3.21, 3.50)	3.33	(1.05, 3.55)

**Table 3:** Proportions endorsing “Some to Very Strong Influence” per Contingency Management Beliefs Questionnaire (CMBQ) item within the General Barriers and Training-related Barriers scales. P-values were derived from exact tests comparing responses from the non-Hispanic White and Hispanic respondents.

	Non- Hispanic White	Hispanic	*p*
General Barriers Subscale			
The research evidence about CM’s effectiveness does not apply to everyday clinic populations.	78.6%	100%	< .001
Clients might sell/trade earned items for drugs.	74.1%	89.7%	.031
A lot of my clients are already abstinent at intake, so they don’t need CM.	43.5%	86.2%	< .001
I find CM distasteful because it is basically paying someone to do what they should do already.	45.9%	89.7%	< .001
CM is expensive (e.g., cost of prizes, vouchers).	70.6%	94.7%	< .001
I am not convinced by the research about CM's effectiveness with substance abusers.	61.2%	82.8%	.006
Providing prizes/vouchers undermines the clients' internal motivation to stay sober.	65.9%	94.8%	< .001
I do not have time to administer vouchers/prizes in a therapy session.	54.1%	79.3%	.002
My clinical experience with recovering substance abusers is more important than any research evidence.	42.4%	82.8%	< .001
Clients will view CM as patronizing.	58.8%	93.1%	< .001
CM interventions create extra work for me.	57.8%	91.4%	< .001
I am worried about what happens once the contingencies are withdrawn.	79.8%	93.1%	.031
CM might cause arguments among clients (e.g., when some get prizes and other do not).	58.3%	89.7%	< .001
I believe it is not right to give rewards for abstinence if clients are not meeting other treatment goals (e.g., group attendance).	58.3%	87.9%	< .001
CM doesn’t address the underlying cause of addiction.	65.5%	82.8%	.035
The community wouldn’t understand (i.e., clinic will look bad for giving rewards to substance abusers).	48.2%	84.5%	< .001
Our clinic rules prevent urine screening.	42.9%	89.3%	< .001
Training-Related Barriers Subscale			
I want more training before implementing CM.	81.2%	94.8%	.023
I don't feel qualified or properly trained to administer CM.	73.8%	78.9%	.551
Currently, no one in my facility has the experience to supervise CM.	73.8%	81.0%	.419
My agency/supervisors/administrators do not support CM (e.g., do not provide training, resources).	67.1%	87.9%	.005
